# FuGePrior: A novel gene fusion prioritization algorithm based on accurate fusion structure analysis in cancer RNA-seq samples

**DOI:** 10.1186/s12859-016-1450-6

**Published:** 2017-01-23

**Authors:** Giulia Paciello, Elisa Ficarra

**Affiliations:** Department of Control and Computer Engineering DAUIN, Politecnico di Torino, C.so Duca degli Abruzzi 24, Turin, 10129 Italy

**Keywords:** Gene fusions, Gene fusion prioritization, Chimeric transcript discovery tools, RNA-sequencing

## Abstract

**Background:**

Latest Next Generation Sequencing technologies opened the way to a novel era of genomic studies, allowing to gain novel insights into multifactorial pathologies as cancer. In particular gene fusion detection and comprehension have been deeply enhanced by these methods. However, state of the art algorithms for gene fusion identification are still challenging. Indeed, they identify huge amounts of poorly overlapping candidates and all the reported fusions should be considered for in lab validation clearly overwhelming wet lab capabilities.

**Results:**

In this work we propose a novel methodological approach and tool named *FuGePrior* for the prioritization of gene fusions from paired-end RNA-Seq data. The proposed pipeline combines state of the art tools for chimeric transcript discovery and prioritization, a series of filtering and processing steps designed by considering modern literature on gene fusions and an analysis on functional reliability of gene fusion structure.

**Conclusions:**

*FuGePrior* performance has been assessed on two publicly available paired-end RNA-Seq datasets: The first by Edgren and colleagues includes four breast cancer cell lines and a normal breast sample, whereas the second by Ren and colleagues comprises fourteen primary prostate cancer samples and their paired normal counterparts. *FuGePrior* results accounted for a reduction in the number of fusions output of chimeric transcript discovery tools that ranges from 65 to 75% depending on the considered breast cancer cell line and from 37 to 65% according to the prostate cancer sample under examination. Furthermore, since both datasets come with a partial validation we were able to assess the performance of *FuGePrior* in correctly prioritizing real gene fusions. Specifically, 25 out of 26 validated fusions in breast cancer dataset have been correctly labelled as reliable and biologically significant. Similarly, 2 out of 5 validated fusions in prostate dataset have been recognized as priority by *FuGePrior* tool.

**Electronic supplementary material:**

The online version of this article (doi:10.1186/s12859-016-1450-6) contains supplementary material, which is available to authorized users.

## Background

The impact of somatic mutations in cancer onset, progression and response to treatment has been widely investigated in the last century [1, 2]. Furthermore, special attention has been devoted to the identification of the so called driver mutations that, differently from passenger mutations, have been found to be responsible for abnormal cell proliferation and cancer development [3]. In an effort to provide complete characterization of the mutational landscape underlying different cancer types, several consortia as The Cancer Genome Atlas (TCGA) [4] or the Breast Cancer Surveillance Consortium (BCSC) [5] have been recently established. These projects benefited of recent advances in genome analysis technologies among which Next Generation Sequencing (NGS).

The analysis of the nowadays available mutational data, confirms the high variability and heterogeneity proper of cancers and cancer subtypes. Furthermore, several mutations have been found to be shared by different neoplasia and only some alterations seem to be disease-specific or even pathognomonic. Among mutations gene fusions can be considered a typical example of pathognomonic alterations. Thus, their identification and characterization have been considered, and still are believed to be fundamental for clinical purposes [6]. As an example, TMPRSS2-ERG fusion has been exploited for prostate cancer screening purposes [7], fusions involving MLL gene have been considered for Acute Myeloid Leukemia (AML) treatment stratification [8], RUNX1-RUNX1T1 fusion has been used for AML diagnosis according to World Health Organization (WHO) classifications [9], and PML-RARA fusion for monitoring minimal residual disease after treatment in adult AML [10].

The emergence of NGS technologies some decades ago, particularly RNA-Sequencing (RNA-Seq), gave a further decisive boost to the comprehension of gene fusion role in cancer. Indeed, differently from previous guided approaches (e.g., banding analyses, fluorescence in situ hybridization, array based-experiments), RNA-Seq allowed to identify fusions in a single experiment without any a priori knowledge on the cytogenetic features of neoplastic cells. The power of this approach becomes evident by considering that the 90% of gene fusions discovered in the last 5 years have been identified by NGS data analyses [6]. Furthermore, clinical detection of gene fusions is progressively shifting towards RNA-Seq assays (e.g., Foundation OneHeme assay) and an increasing number of case studies is reporting on clinical responses of patients treated with drugs after gene fusion detection with such assays [11–14].

These considerations explain the plethora of tools that, since late 2010, have been developed for the detection of gene fusions from RNA-Seq data, most of which rely on paired-end reads [15]. However, as widely discussed in [16], several challenges are still associated to these methods. Besides the considerable amount of time and computational power required for sample processing, these tools output lists of fusions that generally poorly overlap and that are plagued with a huge amount of false positive predictions. Furthermore, the set of filters implemented by these tools to reduce the number of candidates, accounts for reduced sensitivity, potentially causing the loss of real candidates.

To overcome these drawbacks the union of gene fusion lists from different tools should be considered for further analyses. However, temporal and economic constraints make it unfeasible to validate through Polymerase Chain Reaction (PCR) the whole set of fusions from different gene fusion detection tools. Furthermore, the design and implementation of ad-hoc experiments for the functional validation of chimeric transcripts is a complex, expensive and time consuming task that limits once again the number of fusions that can be deepened. In the light of these considerations it seems clear the need for ad-hoc pipelines to shrink down the list of candidates from chimeric transcript discovery tools, thus focusing on a reduced set of highly reliable fusions with a potential driver impact into the disease.

To this aim we propose a novel computational approach and tool named *FuGePrior* for the prioritization of gene fusions from paired-end RNA-Seq data. Specifically, the implemented methodology exploits a set of processing and filtering stages to lower the number of fusions from chimeric transcript discovery tools. These filters have been designed by considering information provided by currently available chimeric transcript discovery tools (e.g., number of supporting reads, gene fusion breakpoints) and modern literature concerning gene fusions. Furthermore, to focus on those fusions with a greater oncongenic driver potential, the driver probability scores provided by two different Machine Learning (ML) algorithms are evaluated in a further filtering stage.

Considering the implementation, *FuGePrior* tool has been developed in Python programming language and can be run downline of all gene fusion detection tools having an output compatible with Pegasus [17] input specifications. Users can easily trigger FuGePrior run to satisfy their requirements as detailed in the “Testing procedure” subsection.

*FuGePrior* has been tested on two publicly available paired-end RNA-seq datasets respectively from Edgren and colleagues [15] and Ren and colleagues [18]. The first one includes four breast cancer cell lines and a normal sample, whereas the second comprises fourteen primary prostate cancer samples and their matched adjacent normal tissues. Both datasets come with a partial in lab validation that has been exploited to prove the strength of the proposed approach in correctly prioritizing real chimeric transcripts.

## Implementation

*FuGePrior* tool consists of a series of filtering and prioritization steps that are applied sequentially to the union list of chimeric candidates from Chimerascan [19], deFuse [20] and a third chimeric transcript discovery tool selected by the user. The unique limitation on the choice of this last algorithm is the compatibility of its output with Pegasus tool [17] input format. The compulsory adoption of both Chimerascan and deFuse has been induced by their wide and well assessed use in current researches and by the goodness of the performance that they achieved on real datasets [16].

**Fig. 1:**
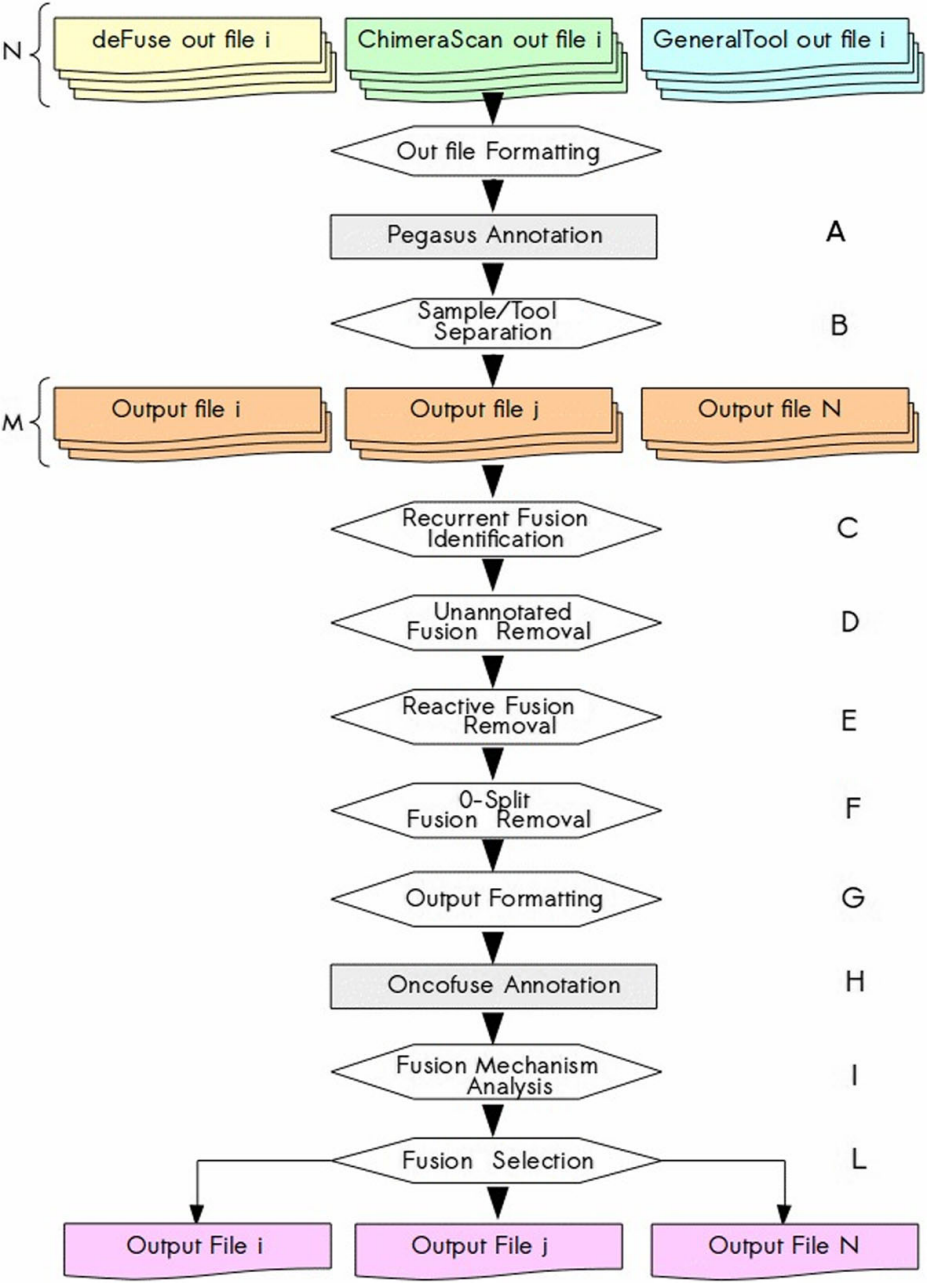
FuGePrior pipeline. The workflow reports on the prioritization and filtering stages implemented by *FuGePrior* tool. Hexagonal shapes account for tasks performed by ad-hoc developed programs, the *grey rectangular* ones refer to those tasks performed by state of the art tools and irregular shapes represent output files. *Yellow, light green and light blue shapes*, report on deFuse, ChimeraScan and MapSplice output respectively. *Orange shapes* identify the intermediate outputs

All the steps constituting *FuGePrior* pipeline are summarized in the scheme of Fig. 1 and detailed in the following. In the workflow, hexagonal shapes account for tasks executed by ad-hoc developed programs, the grey rectangular ones refer to tasks implemented by state of the art tools and irregular shapes represent output files. In details, yellow, light green and light blue shapes report on the N output files from deFuse, ChimeraScan and a third gene fusion detection tool, with N equal to the number of samples under investigation. Orange shapes identify the intermediate outputs, whereas the pink ones account for the final output files. Furthermore, each task is labelled in the diagram and in the text with a progressive upper case letter.

First of all, fusions are annotated using Pegasus tool (***A, first phase***). The input for Pegasus run is constituted by the list of gene fusions identified by the three chimeric transcript discovery tools in the samples under investigation. ChimeraScan and deFuse outputs can be processed by Pegasus without the need for substantial file formatting operations. Conversely, gene fusion data from the third tool has to be opportunely elaborated to be analysed by Pegasus. It is worth noting that this elaboration can be performed on most of chimeric transcript discovery tool outputs, thus allowing FuGePrior to postprocess data from a plethora of gene fusion discovery tools. Details are reported in the “Testing procedure” subsection. By using ENSEMBL gene annotations [21], Pegasus reconstructs the nucleotide sequence of each fusion. This activity is performed by considering, for each fusion, all the transcripts from the two partner genes that account for the identified breakpoints. Thus, each fusion can be described by more than a nucleotide sequence. Based on these sequences, Pegasus assesses the preservation of the reading frame, derives the amino acid sequence of the chimeric protein and evaluates the conservation or loss of the protein functional domains within the partner genes (by interrogating UniProt Web Service [22]). The adoption of Pegasus at the beginning of the pipeline is justified by the need for aggregated and standardized gene fusion information. Indeed, the internal database structure of Pegasus, allows each fusion to be described by the same number and kind of data. This data is partially retrieved from gene fusion detection tool outputs (e.g., number of supporting reads) and partially elaborated after ENSEMBL and UNIPROT database interrogation (e.g., gene fusion sequences, amino acid sequences, reading frame, conserved and lost protein domains). It is worth noting that the common repository embedded in Pegasus, accounts for the identification of fusion candidates shared by more than a sample. This information can be exploited by users to distinguish between the so called private fusions (i.e., fusions occurring in a unique sample) and shared fusions (i.e., fusions occurring in more than a sample).

The aggregated output from Pegasus (a unique file containing all the annotated fusions from different tools in different samples) is then elaborated to label fusions according to the sample (one or more) in which they have been found and the tool (one or more) responsible for their identification (***B***). Since this step, fusions will be analysed in a sample-centered manner. Thus, at the end of *FuGePrior* run, users will be provided with a result file for each of the analysed sample. In these files, each fusion is described by a series of information (e.g., number of supporting reads, driver score probabilities, list of samples in which the fusion has been found) that will make easier the selection of a set of fusions for further in lab investigation.

As pointed out in [16, 23], gene fusion detection tools provide in output poorly overlapping results due to their non inclusive nature. As already mentioned, this is the reason for the adoption of more than a tool for gene fusion discovery and for the choice of considering the union and not the intersection of predictions from different tools. Nevertheless, it is important to annotate fusions identified by more than a tool because they could be with higher probability real fusions. Furthermore, we experimentally proven that a tool can identify the same fusion within a sample by using different supporting reads. Such events are identified and labelled in *FuGePrior* output files (***C***).

Then, those candidates involving unannotated partner genes are removed from the list of fusions reported for each sample (***D***). Indeed, being the function of these genes not assessed yet, it is not possible to hypothesize a role of the fusion into the pathology and to estimate a driver probability for the same.

The identification of gene fusions in non-neoplastic tissues [24–26] led to the design of the next filtering stage since suggesting the existence of fusions that do not have a pathogenetic role. Fusion detection is performed in healthy samples from the same tissue of neoplastic samples by using the tools already exploited for gene fusion discovery in tumor samples (***E***). Those fusions identified in neoplastic samples that are shared by at least a healthy sample are removed from the list of *priority* fusions because with high probability they are not responsible for tumor onset and progression.

Later, fusions supported by at least 1 split read (i.e., reads harbouring within their sequences the fusion breakpoints) are selected from the list of gene fusions (***F***). Indeed, the presence of these reads, by allowing the accurate reconstruction of the gene fusion sequence, accounts for the possibility to validate the fusion throw PCR-based experiments.

The selected fusions are then formatted conveniently to run Oncofuse tool [27] (***G***). By implementing a Naive Bayes Network Classifier, Oncofuse identifies gene fusions that could behave as driver of oncogenic processes and assigns them a driver score probability (***H***). Conversely, Pegasus provides for each fusion a driver score probability that results from a binary classification algorithm using gradient tree boosting. The classifier is trained on a feature space of protein domain annotations and validated tumor fusions (***A, second phase***). Thus, all the fusions are labelled with two scores describing their driver probability.

At the same time, all the fusions selected in (***F***) are further evaluated by considering the biological mechanism underlying their sequences. Specifically, depending on the tool responsible for the detection, the gene fusion consensus sequence or the split reads supporting the fusion are retrieved. For each fusion, four virtual references are reconstructed, according to the breakpoint coordinates reported by chimeric transcript discovery tools. These virtual references account respectively for the retention in the fusion sequence of i) a promoter region in the 5’ gene, and a 3’ end region in the 3’ gene, ii) a promoter region in the 3’ gene, and a 3’ end region in the 5’ gene, iii) a promoter region in both the partner genes and iv) a 3’ end region in both the partner genes. The gene fusion consensus sequence or the split reads supporting the fusion (depending on the tool responsible for gene fusion detection) are then matched against the four different virtual references. This is done to assess which portions of the two partner genes are retained in the fusion (***I***). Consequently, the structural mechanism underlying the observed event and the transcriptional potential of the fusion can be hypothesized. In the following we will define reliable fusions those fusions accounting for the conservation of a promoter region in the 5’ gene and a 3’ end region in the 3’ partner gene, or the conservation of both the promoters in the two fused genes. Indeed, these fusion structures are plausible from a biological view point and could account for high transcription rates.

As last step of the method, fusions with a biologically reliable structure and/or having a Pegasus and/or Oncofuse driver probability higher than a fixed threshold are extracted. These fusions are marked as *priority* since reliable and with a potential role in the pathology (***L***).

## Results

*FuGePrior* tool has been tested on two paired-end RNA-Seq publicly available datasets from Edgren and colleagues [15] and Ren and colleagues [18] respectively. The breast cancer dataset has been downloaded from the Sequence Read Archive (SRA) with accession code SRP003186, whereas the second one from the European Nucleotide Archive (ENA) with accession numbers ERS025221-ERS025248. The first dataset includes four breast cancer cell lines (e.g., MCF-7, KPL-4, BT-474 and SK-BR-3) and a normal breast sample. The latter instead comprises fourteen primary prostate cancer samples and their matched adjacent tissues.

Both datasets have been selected since i) reads in paired-end format allow to run state of the art chimeric transcript discovery tools which outputs constitute the input required to perform *FuGePrior* analysis, ii) they come with a partial in lab validation, thus allowing to make considerations concerning the effectiveness of the proposed approach in prioritizing real fusions, iii) they include samples from healthy tissues that can be exploited to implement *Filter* ***E*** of the proposed pipeline.

Furthermore, one dataset comes from the sequencing of cancer cell lines, whereas the other from the sequencing of primary tumor tissues. We exploited this feature to further discuss *FuGePrior* results on data from different sources.

The following subsections report on the running parameters adopted to analyse breast and prostate cancer datasets and on *FuGePrior* results on the same datasets.

### Testing procedure

Both paired-end RNA-Seq datsets on which we run *FuGePrior* tool have been downloaded in fastq read format. However, read identifiers must be opportunely formatted to perform gene fusion detection. Specifically, *mate_1* and *mate_2* (i.e., the two sequenced ends of a cDNA fragment) need to be labelled with */1* or */2* respectively, according to deFuse specifications. This task has been executed by an ad-hoc developed script.

As highlighted in the “Implementation” section, deFuse and ChimeraScan runs are compulsory to perform *FuGePrior* analysis. Conversely, users are let free to select a third chimeric transcript discovery tool according to their needs. The choice of the third tool is only limited by the compatibility of its output with Pegasus input specifications. We run the latest versions of deFuse (deFuse 0.6.1), ChimeraScan (ChimeraScan 0.4.5) and MapSplice (MapSplice 2.1.8) [28] with default configurations on hg19 reference genome. Furthermore, we triggered MapSplice run in order to report also on the so called well annotated fusions. MapSplice output files have been then formatted in the Pegasus general file format by an ad-hoc developed script. Two additional scripts allowed to adapt deFuse and ChimeraScan output files to Pegasus input requirements. Specifically, deFuse latest version output has been reformatted according to deFuse previous version output (compatible with Pegasus) and ChimeraScan 0-based coordinates have been converted in 1-based coordinates to be comparable with deFuse and MapSplice results. These two scripts are provided to users together with *FuGePrior* code. Gene fusion lists from deFuse, ChimeraScan and Mapslice are then processed by Pegasus (latest version). Pegasus configuration file has been opportunely modified by specifying the sample identifiers and the relative tissue of origin. Results from Pegasus run are then elaborated by *FuGePrior* as detailed in the “Implementation” section. As already mentioned, *FuGePrior* run can be easily and highly customized to answer user needs. First of all, by modifying *FuGePrior* configuration file, users can select which unannotated genes (or not interesting genes) have to be removed from the final list of candidates. We performed the analyses reported in this manuscript by removing all the fusions involving genes which names begin with one of the following strings AC0, AC1, AK, AD0, AL0, AL1, AL5, AL6, AP0, NCRNA, LL22NC, CTC, RNASE, HLA, BC0, AL6, BC1, LOC. Similarly, the tissue of origin of the tumor can be specified in the configuration file. Thus, allowing the generation of an ad-hoc formatted input file for Oncofuse run. EPI (i.e., epithelial origin), HEM (i.e., hematological origin), MES (i.e., mesenchymal origin) and AVG (i.e., average expression, if tissue source is unknown) are the labels that users can specify in *FuGePrior* configuration file. Our experiments have been performed by specifying MES string in the configuration file and using Oncofuse latest version (Oncofuse 1.0.9). *FuGePrior* evaluates the biological mechanism at the basis of gene fusion structure by reconstructing four different virtual references that account for the retention in the gene fusion of different portions of the partner genes. These virtual references are later matched against ChimeraScan split reads and deFuse/MapSplice consensus sequences to determine the gene fusion structure. Users can specify the length of the four reconstructed virtual references and the minimum overlap between reads/consensus sequence (depending on the tool) and virtual reference, required to label the fusion with a specific gene structure information. The analyses performed on breast and prostate cancer datasets consider a virtual reference length of 30 bp and a minimum overlap to correctly label a fusion equal to 15 bp. Finally, users can fix the driver score probability threshold exploited by *Filter* ***L***. We selected 0.7 as threshold in the proposed analyses.

### Breast cancer dataset

ChimeraScan detected 55, 27, 197 and 132 fusions in MCF-7, KPL-4, BT-474 and SK-BR-3 cell lines, respectively. Conversely, deFuse found 39, 42, 319 and 231 chimeric transcripts in the same cell lines. A very reduced number of fusions is instead reported by MapSplice in the different cell lines. This number ranges from 4 fusions in KPL-4 cell line to 36 in BT-474. Furthermore, the same tools identified 41, 60 and 1 fusions in the normal breast sample.

**Fig. 2:**
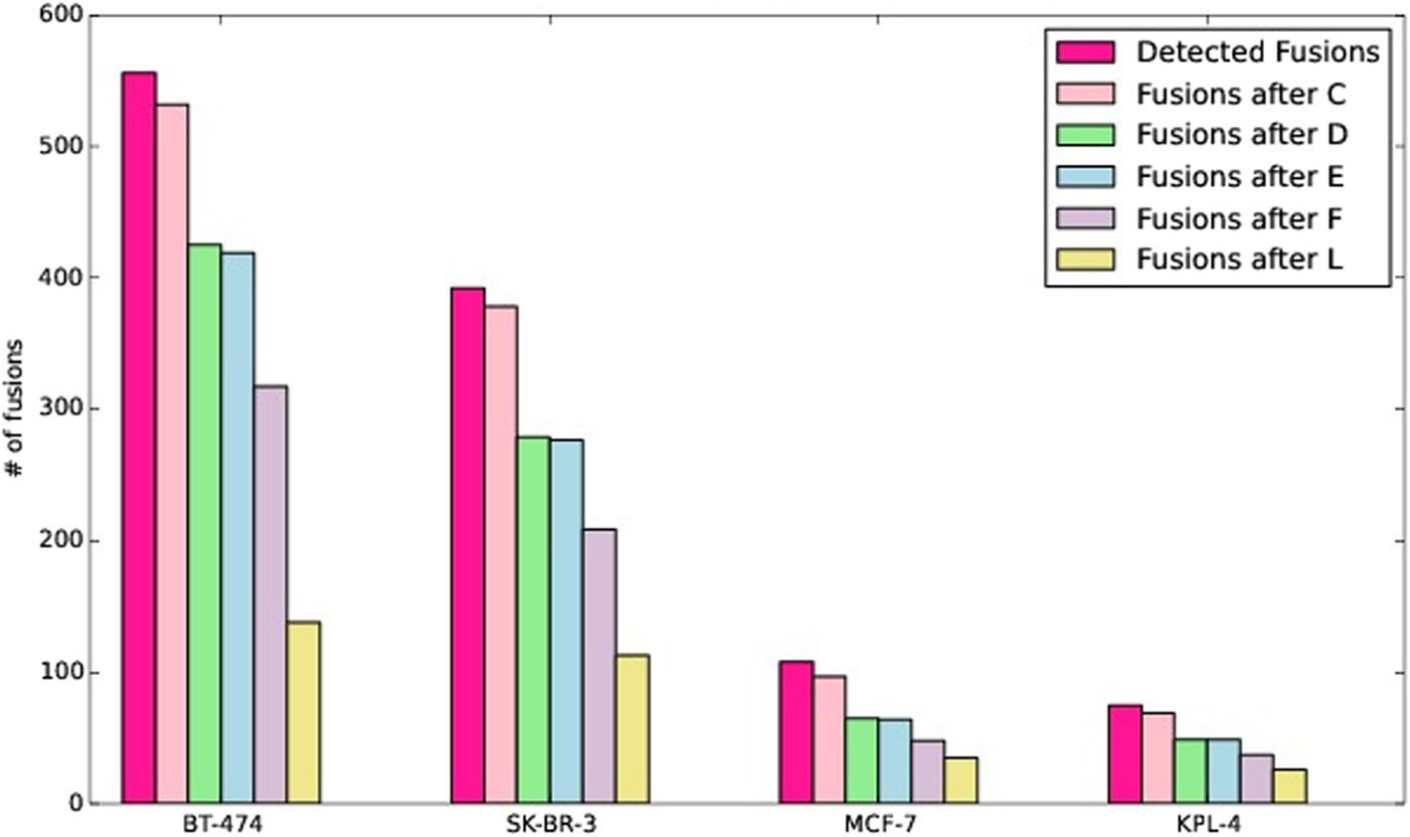
FuGePrior filtering in Breast Cancer dataset. Each group of bars reports, for the different breast cancer cell lines on x-axis, on the number of fusions retained after the application of the different filtering stages implemented by *FuGePrior* pipeline

The barchart of Fig. 2 describes, for the breast cancer cell lines under investigation, the impact of the filtering stages implemented within *FuGePrior* on the number of retained gene fusions.

**Fig. 3:**
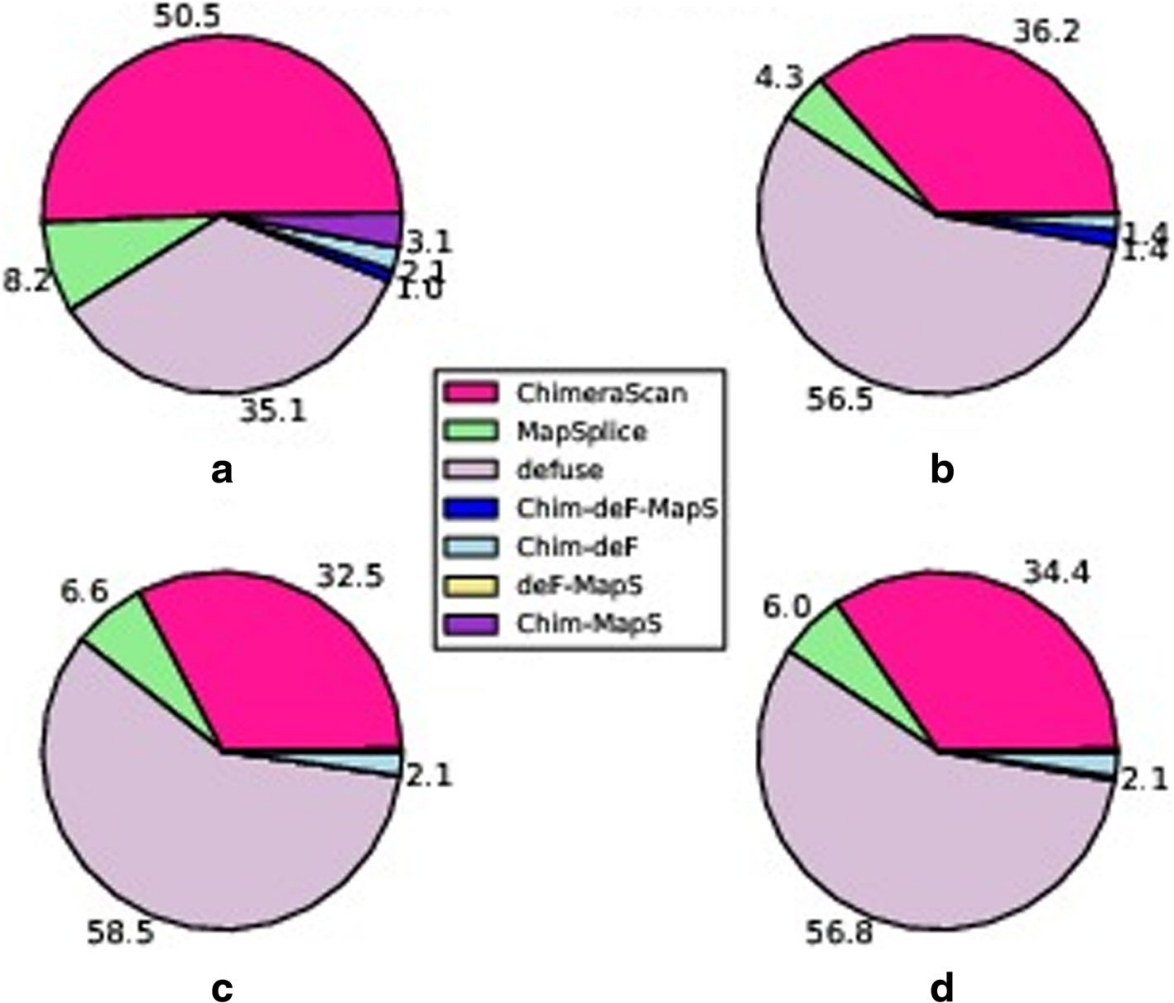
Consensus among tools in Breast Cancer dataset. Subfigures 3a, 3b, 3c and 3d report for MCF-7, KPL-4, SK-BR-4 and BT-474 respectively on the percentage amounts of fusions identified by the three considered gene fusion discovery tools or combinations among them

The collapsing of the fusions performed by *Filter* ***C*** accounted for a reduction in the amount of reported fusions that varies from a minimum of 2.8% in SK-BR-3 to a maximum of 7.6% in MCF-7. We identified 2 fusions (BCAS4/BCAS3 and UNC45B/DLG2), 1 fusion (UNC45B/DLG2), 3 fusions (MT-ND6/MT-ATP-6, KLF15-AL121656.4 and THRA-AC090627.1) and 1 fusion (UNC45B/DLG2) reported by deFuse in MCF-7, KPL-4, BT-474 and SK-BR-3 cell lines respectively with different supporting reads. None chimeric transcripts are instead reported by ChimeraScan and MapSplice as supported by different reads. Furthermore, we deeply investigated the agreement among tools on gene fusion discovery, pointing out a poor well known overlap among their results. Specifically, only 2, 1, 11 and 8 fusions are identified by both ChimeraScan and deFuse in MCF-7, KPL-4, BT-474 and SK-BR-3 respectively. 0, 0, 1 and 0 fusions are reported by deFuse and MapSplice in the same cell lines. 3, 0, 1 and 1 fusions have been found by both ChimeraScan and MapSplice. Finally, only 1 (BCAS4/BCAS3), 1 (BSG/NFIX), 2 (STX16/RAE1, RAB22A/MYO9B) and 0 fusions have been reported by all the tools within the considered breast cancer cell lines. Figure 3 shows the percentage numbers of fusions reported, in the different cell lines, by the deFuse, ChimeraScan and MapSplice tools or combinations among them. For visualization reasons, values are rounded to the first decimal place.

**Fig. 4:**
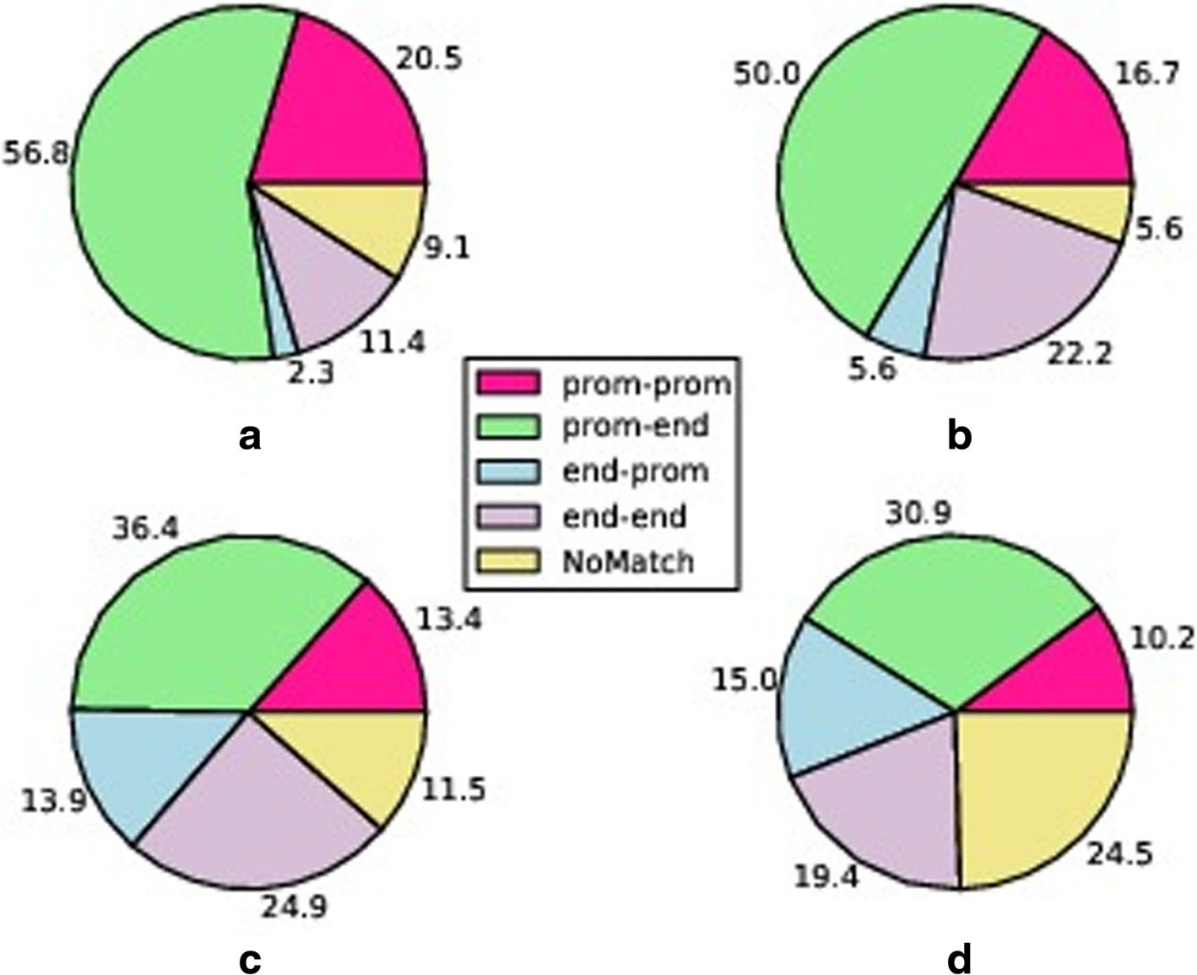
Gene fusion structures in Breast Cancer dataset. Subfigures 4a, 4b, 4c and 4d report for MCF-7, KPL-4, SK-BR-4 and BT-474 respectively on the average percentages of fusions characterized by the five different fusion structures we investigated after *FuGePrior* Filter F application

The removal of fusions involving unannotated partner genes (*Filter* ***D***) produced a conspicuous decrease in the number of events to be considered in the next steps of the workflow. Indeed, 32 (32.9% of reduction), 20 (28.9% of reduction), 107 (20.1% of reduction) and 99 (26.2% of reduction) fusions have been respectively deleted from the set of chimeras in MCF-7, KPL-4, BT-474 and SK-BR-3 cell lines. Results are little impacted by *Filter* ***E***. Indeed, this filter accounted for the removal of 1, 0, 6 and 2 gene fusions in MCF-7, KPL-4, BT-474 and SK-BR-3 cell lines respectively. Conversely, a huge amount of fusions is filtered out because not supported by split reads (Filter **F**). Indeed, only 48, 37, 317 and 209 fusions are supported by at least a split read in MCF-7, KPL-4, BT-474 and SK-BR-3 cell lines. Furthermore, we investigated the amount of fusions that, after *Filter* ***F*** application, presents a reliable (according to our previous definition) gene fusion structure. We found that 25 out of 48 fusions in MCF-7 cell line, 18 out of 37 in KPL-4, 97 out of 317 in BT-474 and 76 out of 209 in SK-BR-3 account for the retention of a promoter sequence in the 5’ partner gene and for a 3’ end region in the 3’ gene. Furthermore, 9, 6, 32 and 28 fusions in the same breast cancer cell lines retained a promoter region in both the partner genes. To be as thorough as possible, we report in the piecharts of Fig. 4 on the percentage number of fusions characterized by the different analysed gene fusion structures. For visualization reasons, values are rounded to the first decimal place. In the legend, *prom-end* and *prom-prom* refer to those fusions that we defined as biologically reliable since accounting for a promoter sequence in the 5’ partner gene and for a 3’ end region in the 3’ partner gene or for both promoters retained in the fusion. Conversely, *end-prom* label refers to fusions characterized by a promoter sequence in the 3’ partner gene and a 3’ end region in the 5’ partner gene. *end-end* label refers to fusions that retain a 3’ gene end region in both partner genes. Finally, *NoMatch* label is relative to fusions for which we did not find a match on the four reconstructed virtual references.

The analysis of Pegasus and Oncofuse driver scores (*Filter* ***A, second phase*** and *Filter* ***H***) led to the identification of 6, 4, 28 and 27 fusions in MCF-7, KPL-4, BT-474 and SK-BR-3 cell lines for which one or both tools provided a driver score greater than 0.7. Among these fusions, 6 out of 6 in MCF-7, 3 out of 4 in KPL-4, 21 out of 28 in BT-474 and 23 out of 27 in SK-BR-3 have a biologically reliable structure.

By implementing *Filter* ***L***, fusions with a driver score greater than 0.7 and/or characterized by a plausible biological mechanism are selected and marked as reliable and with a potential role into the pathology. As a result, 34, 25, 137 and 111 fusions respectively in MCF-7, KPL-4, BT-474 and SK-BR-3 belong to the final list of *priority* fusions. These values account for a reduction in the number of fusions output of chimeric transcript discovery tools equal to 66.6, 64.4, 75 and 70.9% in MCF-7, KPL-4, BT-474 and SK-BR-3 respectively. For completeness we report in Additional file 1: (S1) on the consensus among chimeric transcript discovery tools pointed out in the final list of *FuGePrior* *priority* fusions.

The lack of not-synthetic datasets with a complete gene fusion validation, makes the assessment of the performance of gene fusion detection and prioritization tools very challenging, in terms of both sensitivity and specificity. However, for some datasets as the one under investigation, a partially validation of the fusions is available. Specifically, the study by Edgren reports on 3, 3, 11 and 10 fusions identified respectively in MCF-7, KPL-4, BT-474 and SK-BR-3 cell lines. In absence of a full validated dataset, the best that can be done to evaluate results from the novel in-silico procedure we propose, consists in comparing gene fusions that we found to be *priority* after *FuGePrior* analysis with those previously validated by Edgren and colleagues.

These results are reported in Additional file 1: (S2). Concerning MCF-7 all the 3 validated fusions have been prioritized by the proposed approach. Specifically, all the fusions are characterized by a biologically reliable structure.

All the validated fusions from KPL-4 cell line, passed the filtering stages implemented in the proposed pipeline. 2 out of 3 fusions account for both a reliable biological structure and high driver scores, 1 out of 3 fusion satisfies only the reliability criterion.

Ten out 11 validated fusions in BT-474 cell line are reported as output of the implemented methodology. In particular, 5 have been retained since satisfying both the rules (driver scores and biological mechanism), whereas 5 because characterized by a biologically reliable structure. The only missed fusion has been removed since not supported by split reads.

Finally, 9 out 10 chimeric transcripts have been detected as *priority* by our methodology in SK-BR-3 cell line. 3 have high driver scores and plausible structures, whereas the remaining satisfy the second criterion. Only 1 validated fusion has not been reported since not identified by gene fusion detection tools.

### Prostate cancer dataset

**Fig. 5:**
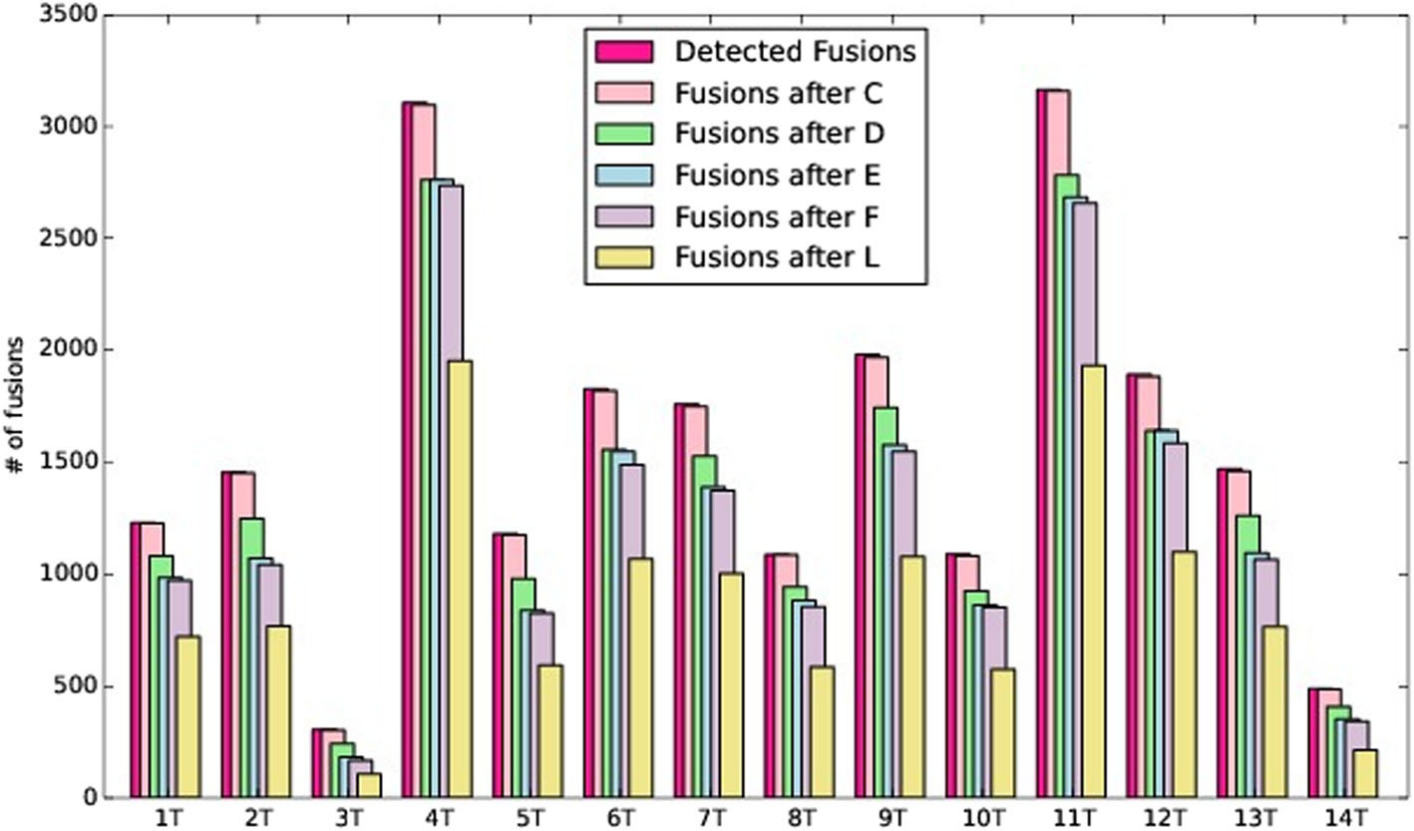
FuGePrior filtering in Prostate Tumor dataset. Each group of bars reports, for the different prostate cancer samples on x-axis, on the number of fusions retained after the application of the different filtering stages implemented by *FuGePrior* pipeline

ChimeraScan, deFuse and MapSplice reported on an average number of fusions in the 14 samples from Ren dataset respectively equal to 91, 1465 and 11. Even in this dataset, chimeric transcript discovery tools rarely agree on the identified gene fusions. In details, the mean number of fusions identified by both ChimeraScan and deFuse is equal to 1, whereas no shared fusions are in average reported for the other combinations of algorithms. The complete analysis is reported in Additional file 1: (S3). The barcharts of Fig. 5 describe, for the different tumor samples included in prostate cancer dataset, the impact of the filtering stages implemented by *FuGePrior* on the number of retained gene fusions. *Filter* ***C*** is responsible for the removal of a mean number of fusions equal to 7 across the 14 samples under investigation, with a maximum of 14 fusions discarded in Sample 12T. The impact of *Filter* ***D*** for unannotated fusion removal, varies depending on the considered sample with a minimum reduction observed in Sample 4T (10.8%) and a maximum occurring in Sample 5T (16.5%).

**Fig. 6:**
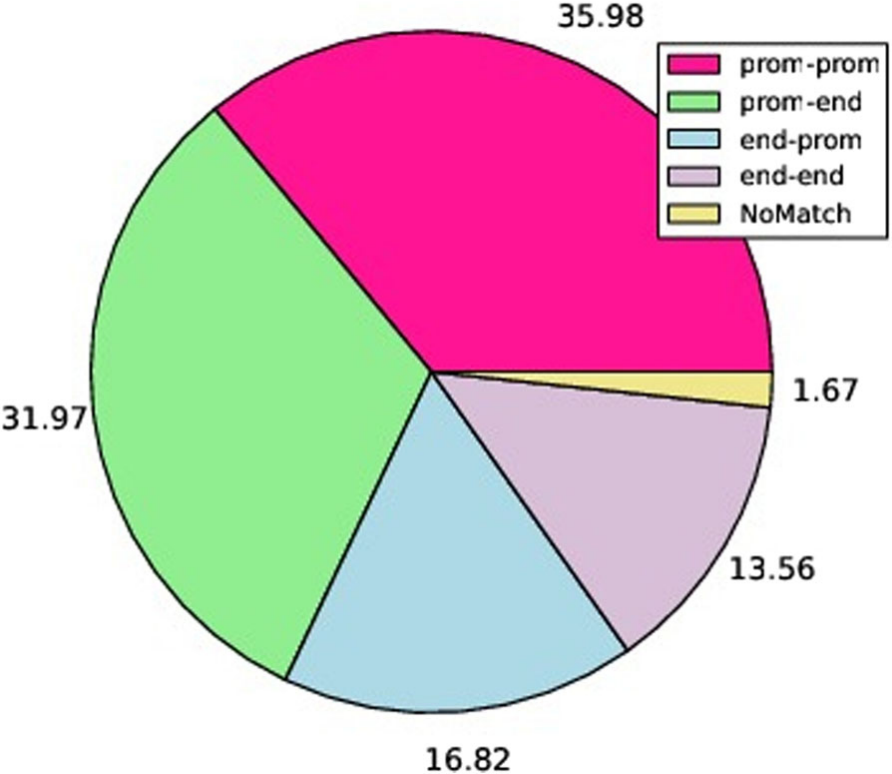
Gene fusion structures in Prostate Tumor dataset. The piechart reports on the average percentages of fusions characterized by the five different fusion structures we investigated after *FuGePrior* Filter F application

Ren dataset includes, for each tumor sample, the adjacent normal tissue, allowing the application of *Filter* ***E*** that acts by removing fusions shared by reactive samples. We observed a maximum percentage decrease in the number of fusions (24.7%) in Sample 3T, that shares 60 fusions with the adjacent normal tissue. The removal of fusions not supported by split reads, performed by *Filter* ***F***, is once a time more evident in Sample 3T with a percentage reduction equal to 8.7%. Furthermore, we investigated the biological mechanism at the basis of the fusions retained after *Filter* ***F*** application. The piechart of Fig. 6 reports on the average percentages of fusions characterized by the different investigated gene fusion structures. As it is possible to note from the graph a not negligible percentage of fusions is characterized by a biologically not reliable gene structure.

The greater impact in gene fusion number reduction is produced by *Filter* ***L***. As already explained, the filter works by evaluating the driver score probabilities provided by Oncofuse and Pegasus tools and the biological mechanism at the basis of the fusion. Its application accounted for an average percentage reduction in the number of output fusions of about 46.4% across the considered samples. For completeness, we report in Additional file 1: (S3) on the agreement among ChimeraScan, deFuse and MapSplice tools in *FuGePrior* output fusion identification.

Finally, we focused on the 5 validated gene fusions from Ren and colleagues to check for their presence in the final list of *priority* gene fusions from *FuGePrior*. Results are reported in Additional file 1: (S4). In detail, USP9Y-TTTY15 fusion, identified by Ren in Samples 4T and 6T and 12T is not present in *FuGePrior* output relative to these samples. This is because none of the chimeric transcript discovery tools reported on it. It is worth noting that *FuGePrior* prioritized 3 different reciprocal gene fusions (TTTY15-USP9Y). Two of them occurring in Sample 4T and the other in 6T with different breakpoints. USP9Y-TTTY15 fusion has been identified for the first time in the experiments performed by Ren and its occurrence in several samples allows to hypothesize a role of the fusion in cancer development. The well known prostate cancer fusion TMPRSS2-ERG has been validated in Samples 1T, 5T and 13T. *FuGePrior* correctly prioritized this fusion in the same samples with breakpoints on hg19 corresponding respectively for 5’ gene and 3’ gene to chr21: 42880008 and chr21: 39817544. The fusion has been identified by both ChimeraScan and deFuse in Sample 5T, whereas only ChimeraScan reported on it in Samples 1T and 13T. The fusion involving TMPRSS2 and ERG genes occurr between exon 1 of the first partner and exon 4 of the second. However, note that several other breakpoints on these genes have been recently described [29]. Furthermore, additional analyses performed by Ren and colleagues on 54 prostate tumor samples confirmed the presence of TMPRSS2-ERG fusion in Chinese population at lower frequency (about 20%) with respect to that observed in Caucasian patients [30]. RAD50-PDLIM4 fusion has been found, confirming Ren results, in Sample 10T with fusion breakpoints on hg19 corresponding to chr5:131945088 and chr5:131598302. The three fusion discovery tools agreed on its identification within Sample 10T. The last two validated fusions, SDK1-AMACR in Sample 7T and CTAGE5-KHDRBS3 in Sample 10T, are not reported as output of *FuGePrior* run due to the fact that they are not present in the output from chimeric transcript discovery tools. Concerning CTAGE5-KHDRBS3, SDK1-AMACR, and RAD50-PDLIM4 gene fusions Ren proven their occurrence also in the additional 54 prostate tumor samples analysed (with percentages ranging from 24 to 37%). Additional evidences for their occurrence in prostate Chinese tumor samples are discussed in [31].

## Discussion

Chimeric transcript discovery tools generally provide as output huge lists of poorly overlapping gene fusions. Higher the coverage of the samples under investigation, higher the number of reported candidate fusions. We considered in this work two datasets. The first from breast cancer cell lines characterized by quite reduced coverage (number of reads ranging from a minimum of 13600332 reads in KPL-4 cell line to a maximum of 42861028 in SK-BR-3), and the other from prostate primary tumor samples that includes about 1860097798 reads with a maximum number of reads equal to 150304440 in Sample 13T and a minimum of 43908581 reads in Sample 3T. The not comparable dimensions of the two datasets explain the large difference in the number of fusions reported by chimeric transcript discovery tools in the considered datasets. Furthermore, it is worth noting that the very reduced number of fusions identified by MapSplice was expected. Indeed, we pointed out the same trend in analyses we performed on private datasets from different pathologies. However, for both datasets, the huge number of fusion candidates from chimeric transcript discovery tools makes unfeasible the in lab validation of all these predictions, thus calling for ad-hoc strategies to shrink down the number of fusions, focusing on a reduced set of highly reliable fusions.

As pointed out in the “Results” section, chimeric transcript discovery tools rarely agree on the provided predictions. Furthermore, in few cases they identify the same fusion using different reads. This explains the derisory impact of *Filter* ***C*** in reducing fusion numbers.

A more evident impact in gene fusion removal is due to *Filter* ***D*** implementation. The high number of fusions involving unannotated genes leads to reflect on the fact that there is still much to be done. Indeed, even if these genes are nowadays only partially known, they could have an active role in cancer processes. *Filter* ***E*** application produced different results in the two considered datasets. Indeed, it is responsible for an average reduction in the number of fusions equal to 0.9% and 9% in breast cancer cell lines and primary prostate tumor data respectively. These results can be explained by the fact that the reactive samples of the second dataset are “more specific” since match the adjacent prostate tumor tissue. Thus, they could account for higher similarity with tumor samples and so for a greater number of fusions shared with them. The removal of those fusions not supported by split reads (*Filter* ***F***) produced a relevant decreasing in the number of output candidates. All the removed fusions are from ChimeraScan tool, since deFuse and MapSplice do not report predictions with 0 split reads. The absence of split reads accounts for the incapability in reconstructing a fusion sequence from chimeric transcript detection tool outputs unless additional mapping and processing steps. However, it should be considered that these additional steps are prone to errors and could lead to the identification of false positive fusions.

The analysis of gene fusion structure (***I***) pointed out, in both the datasets, a not net prevalence of transcripts having a biologically reliable configuration. Indeed, as summarized in Figs. 4 and 6, respectively for breast and prostate datasets, a relevant number of fusions from chimeric transcript discovery tools accounts for the structures we labelled as *end-end* and *end-promoter*. This finding should be deeply investigated with in lab experiments to assess if these fusions are false positive predictions or not and, in case of positive results, to evaluate the transcriptional potential of such aberrations. As expected, Oncofuse and Pegasus tools (***A, second phase*** and ***H***) produced little overlapping results because of the different classification methods they adopt. For instance, they agreed in the assignment of a driver score greater than 0.7 for 1, 2, 4, and 2 fusions in MCF-7, KPL-4, BT-474 and SK-BR-3 cell lines respectively. On average, 16 fusions are prioritized with a driver score greater than 0.7 by both Oncofuse and Pegasus in the 14 Samples from prostate cancer. We considered the union of high scored fusions from the tools to be as exhaustive as possible. Finally, the choice to focus in the last phase of the pipeline, on the union set of high scored and biologically reliable fusions (*Filter* ***L***), has been made, once a time, to be conservative, thus avoiding loss of sensitivity. Indeed, in previous analyses on Burkitt lymphoma samples we validated through PCR experiments fusions characterized by a non reliable fusion structure but having high driver scores, while in AML samples we were able to confirm in lab fusions with a reliable structure but low driver scores.

Concerning the validated fusions, it is worth noting that only 2 out 3 fusions in MCF-7 have been identified by two tools, whereas none has been reported by all chimeric transcript discovery tools. 1 out of 3 fusions in KPL-4 has been reported by all the tools, 1 by two tools. 2 out of 10 and 2 out of 9 fusions in BT-474 and SK-BR-3 respectively have been identified by two tools. A unique fusion in both these last cell lines has been reported by all the tools. Relatively to the two validated fusions confirmed by *FuGePrior* in prostate dataset, TMPRSS2-ERG has been identified by both ChimeraScan and deFuse in Sample 5T, whereas only ChimeraScan reported on it in Samples 1T and 13T. Furthermore, RAD50-PDLIM4 fusion has been found in Sample 10T by all the fusion discovery tools. These results confirm the need for considering the output from more than a tool when performing gene fusion discovery. Furthermore, the presence of validated fusions having only 1 supporting split read confirms the importance of avoiding filtering stages to remove chimeric transcripts supported by few reads.

To summarize, *FuGePrior* analysis accounted for a reduction in the number of fusions from chimeric transcript discovery tools of 66.6, 64.4, 75 and 70.9% respectively in MCF-7, KPL-4, BT-474 and SK-BR-3 cell lines. An average reduction in the number of fusions equal to 46.4% has been instead reported for prostate cancer dataset. Furthermore, 25 out of the 27 breast cancer fusions previously in lab confirmed, have been correctly labelled as *priority* by our pipeline. It is worth noting that 1 of the 2 remaining fusions was not detected by chimeric transcript discovery tools and then it was not given in input to the proposed pipeline. Thus, in synthesis, the proposed method labelled as *priority* 25 out of 26 validated fusions. Similarly, 2 out of 5 validated fusions in prostate cancer have been correctly prioritized by *FuGePrior*. Even in this case the other 3 fusions were not reported by chimeric transcript discovery tools.

## Conclusions

In this work, we propose a methodological approach and tool named *FuGePrior* to shrink down the union set list of candidates from different chimeric transcript discovery tools, thus focusing on a reduced number of reliable fusions with a potential role in the pathology. This novel pipeline includes a series of modules and filtering steps designed by considering both chimeric transcript discovery and annotation tool outputs and state of the art literature concerning gene fusions. *FuGePrior* is implemented in Python programming language and its run can be widely triggered by users depending on their needs. The proposed methodology has been tested on two paired-end RNA-Seq publicly available datasets from breast cancer cell lines and prostate primary tumors. Results accounted for a reduction in the number of fusions output of chimeric transcript discovery tools that ranges from 64.4 to 75% in breast cancer dataset and from 37 to 65% in the prostate one. Furthermore, both datasets come with a partial validation that allowed us to assess the performance of the proposed approach in correctly prioritizing real gene fusions. Specifically, 25 out of 27 validated fusions have been correctly prioritized in breast cancer dataset. Furthermore, 2 out of 5 validated fusions in prostate tumor dataset have been reported as output of *FuGePrior* run. It is worth noting that globally 4 out of 5 validated fusions that have not been prioritized by *FuGePrior* were not given in input to the pipeline because not identified by chimeric transcript discovery tools. Analyses performed on private AML, Acute Lymphoblastic Leukemia (ALL), Burkitt Lymphoma and Mastocytosis samples confirmed the great potential of this methodological approach in providing biologists with a limited number of reliable fusions.

## Availability and requirements

**Project name:** FuGePrior**Home page:** https://philae.polito.it/paciello/FuGePrior/ **Operating system:** Linux**Programming language:** Python, bash**License:** Freely available for academic purposes.

## Additional file

Additional file 1 is a pdf file reporting on supplementary analyses performed to evaluate *FuGePrior* performance. Furthermore, additional tables and figures are presented in this document. (PDF 171 kb)

## Abbreviations

ALL: Acute Lymphoblastic Leukemia
AML: Acute Myeloid Leukemia
BCSC: Breast Cancer Surveillance Consortium
ENA: European Nucleotide Archive
ML: Machine Learning
NGS: Next Generation Sequencing
PCR: Polymerase Chain Reaction
RNA-Seq: RNA-Sequencing
SRA: Sequence Read Archive
TCGA: The Cancer Genome Atlas
WHO: World Health Organization
