# Supplementary material for: FuGePrior: A novel gene fusion prioritization algorithm based on accurate fusion structure analysis in cancer RNA-seq samples
Source: BMC Bioinformatics. 2017 Jan 23;18:58. doi: 10.1186/s12859-016-1450-6 (PMC5260008; doi:10.1186/s12859-016-1450-6)
Supplement: Additional file 1 — is a pdf file reporting on supplementary analyses performed to evaluate FuGePrior performance. Furthermore, additional tables and figures are presented in this document. (PDF 171 kb) [file 12859_2016_1450_MOESM1_ESM.pdf]

Additional File for
"FuGePrior: A novel gene fusion prioritization algorithm based on accurate fusion structure analysis in cancer RNA-seq sample"

Giulia Paciello[1], and Elisa Ficarra[1]

[1]*Dept. of Control and Computer Engineering DAUIN, Politecnico di Torino, C.so Duca degli Abruzzi 24, Turin, 10129, Italy*

(Dated: December 15, 2016)

S1

As widely discussed in the manuscript, gene fusion discovery tools generally output fusion lists that poorly overlap. We further investigated this well known drawback of chimeric transcript discovery algorithms by evaluating the agreement among ChimeraScan, deFuse and MapSplice tools, on the fusions reported as output of *FuGePrior* run. The piecharts of Figure S1 report for MCF-7, KPL-4, SK-BR-4 and BT-474 breast cancer cell lines respectively, on the percentages of gene fusions detected by the three considered gene fusion discovery tools or combinations among them in FuGePrior output. For visualization issues, values are rounded to the first decimal place. The most of fusions in all the cell lines come from deFuse, followed by ChimeraScan. 2,1,8,10 fusions have been identified in the different cell lines by both deFuse and ChimeraScan. Conversely, a negligible consensus has been pointed out for the other combinations of algorithms. Moreover only 1, 1, 0 and 2 fusions have been detected by all the tools.

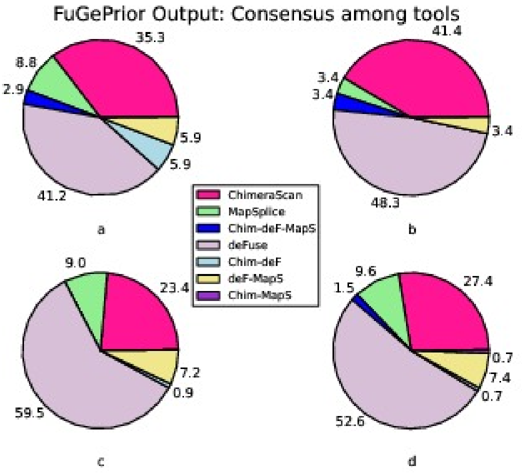

FIG. S1: **Consensus among tools in Breast Cancer dataset**. Subfigures 1a, 1b, 1c and 1d report respectively for MCF-7, KPL-4, SK-BR-4 and BT-474 breast cancer cell lines on the percentages of gene fusions detected by the three considered gene fusion discovery tools or combinations among them in *FuGePrior* output.

S2

Table S1 reports, for the different Breast Cancer cell lines of column 1, on the validated gene fusions. Specifically, in the different columns, from column 2, are indicated the name of the partner genes involved in the fusion, the driver scores provided by Pegasus and Oncofuse tools for the fusion, the criterion satisfied in the last filtering step of the proposed pipeline, and the motivation for their absence as output of the implemented pipeline. Note that the *"-"* symbol in *Oncofuse DS* column accounts for no score reported by the tool.

Cell Line	5' Gene	3' Gene	Pegasus DS	Oncofuse DS	DS >0.7	Reliability	Notes
MCF-7	BCAS4	BCAS3	0.0002	0.0053		X	
	ARFGEF2	SULF2	0.0002	-		X	
	RPS6KB1	TMEM49	0.0002	0.6784		X	
KPL-4	BSG	NFIX	0.0002	0.0119		X	
	PPP1R12A	SEPT10	0.9993	0.9316	X	X	
	NOTCH1	NUP214	0.9650	0.9287	X	X	
BT-474	ACACA	STAC2	0.5414	0.1468		X	
	RPS6KB1	SNF8	0.9964	0.9990	X	X	
	VAPB	IKZF3	0.0002	0.9980	X	X	
	ZMYND8	CEP250	0.0004	0.1472		X	
	RAB22A	MYO9B	0.0004	0.9345	X	X	
	SKA2	MYO19	0.9894	0.3710	X	X	
	DIDO1	KIAA0406	0.1392	-		X	
	STARD3	DOK5	0.9957	0.0053	X	X	
	LAMP1	MCF2L	0.0005	0.5316		X	
	GLB1	CMTM7	0.0084	0.0139		X	
	CPNE1	PI3					No Split Reads
SK-BR-3	TATDN1	ENSG00000236127	0.0086	0.0054		X	
	CSE1L	KCNB1	0.0002	0.0005		X	
	RARA	PKIA	0.0002	0.0005		X	
	ANKRD17	PCDH1	0.9932	0.1138	X	X	
	CCDC85C	SETD3	0.9894	0.3577	X	X	
	SUMF1	LRRFIP2	0.9960	0.0201	X	X	
	WDR67	ZNF704	0.0003	0.3590		X	
	CYTH1	EIF3H	0.4661	-		X	
	DHX35	ITCH	0.0003	0.0145		X	
	NFS1	PREX1					Not found by chimeric transcript discovery tools

TABLE S1: **Validated gene fusions in Breast Cancer cell lines.** The table reports, for the different cell lines of column 1, on the name of the partner genes involved in the fusion, the driver scores provided by Pegasus and Oncofuse tools for the fusion, the criterion satisfied in the last filtering step of the proposed pipeline, and the motivation of their absence as output of the implemented pipeline.

S3

Even in prostate cancer dataset, we observed a very reduced agreement among gene fusion discovery tools. Subfigure 1a reports on the average percentage amounts of fusions from different tools or combinations among them in *FuGePrior* input. The most of fusions have been detected by deFuse. This algorithm reported an average of 1465 fusions across the fourteen considered samples. Conversely, ChimeraScan and MapSplice accounted for an average number of reported fusions equal to 91 and 11. The three tools rarely agreed on predictions. Indeed, we observed an average number of shared fusions slightly greater than 1 only when considering fusions common to deFuse and ChimeraScan. Similar considerations can be done relatively to *FuGePrior* output as highlighted in Subfigure 1b.

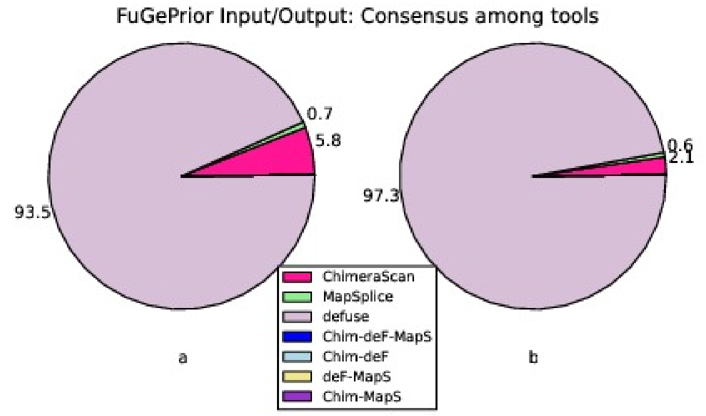

FIG. S2: **Consensus among tools in Prostate Cancer dataset**. Subfigure 1a and 1b report respectively for *FuGePrior* input and output on the average percentages of fusions detected by the three considered gene fusion discovery tools or combinations among them in prostate cancer dataset.

S4

Table S2 reports, for the different Prostate Cancer samples of column 1, on the validated gene fusions. Specifically, in the different columns, from column 2, are indicated the name of the partner genes involved in the fusion, the driver scores provided by Pegasus and Oncofuse tools for the fusion, the criterion satisfied in the last filtering step of the proposed pipeline, and the motivation for their absence as output of the implemented pipeline. Note that the *"-"* symbol in *Oncofuse DS* column accounts for no score reported by the tool.

Sample	5' Gene	3' Gene	Pegasus DS	Oncofuse DS	DS >0.7	Reliability	Notes
1T	TMPRSS2	ERG	0.5826	-		X	
4T	USP9Y	TTTY15					Not found by chimeric transcript discovery tools
5T	TMPRSS2	ERG	0.7202	-	X	X	
6T	USP9Y	TTTY15					Not found by chimeric transcript discovery tools
7T	SDK1	AMACR					Not found by chimeric transcript discovery tools
10T	RAD50	PDLIM4	0.0002	0.9998	X	X	
	CTAGE5	KHDRBS3					Not found by chimeric transcript
12T	USP9Y	TTTY15					Not found by chimeric transcript discovery tools
13T	TMPRSS2	ERG	0.8591	-	X	X	

TABLE S2: **Validated gene fusions in Prostate Cancer Samples.** The table reports, for the different cell lines of column 1, on the name of the partner genes involved in the fusion, the driver scores provided by Pegasus and Oncofuse tools for the fusion, the criterion satisfied in the last filtering step of the proposed pipeline, and the motivation of their absence as output of the implemented pipeline.
